# Antithrombotic Treatment of Embolic Stroke of Undetermined Source RE-SPECT ESUS Elderly and Renally Impaired Subgroups

**DOI:** 10.1161/STROKEAHA.119.028643

**Published:** 2020-05-14

**Authors:** Hans-Christoph Diener, Ralph L. Sacco, J. Donald Easton, Christopher B. Granger, Michal Bar, Richard A. Bernstein, Michael Brainin, Martina Brueckmann, Martina Brueckmann, Lisa Cronin, Geoffrey Donnan, Zuzana Gdovinová, Claudia Grauer, Eva Kleine, Timothy J. Kleinig, Philippe Lyrer, Sheila Martins, Juliane Meyerhoff, Truman Milling, Waltraud Pfeilschifter, Sven Poli, Michal Reif, David Z. Rose, Daniel Šaňák, Wolf-Rüdiger Schäbitz

**Affiliations:** Faculty of Medicine, Institute for Medical Informatics, Biometry and Epidemiology, University Duisburg-Essen, Germany;; Clinical and Translational Science, Miller School of Medicine, University of Miami, FL;; Department of Neurology, University of California, San Francisco;; Duke Clinical Research Institute, Duke University Medical Center, Durham, NC;; Department of Neurology, University Hospital Ostrava, Ostrava-Poruba-Poruba, Czech Republic;; Department of Neurology, Northwestern University, Chicago, IL;; Department of Neurosciences and Preventive Medicine, Danube University Krems, Krems an der Donau, Austria;; Metabolism Medicine, Boehringer Ingelheim International GmbH, Germany;; Faculty of Medicine Mannheim of the University of Heidelberg, Germany;; Cardiometabolic Medicine, Boehringer Ingelheim Ltd, Burlington, ON, Canada;; Department of Neurology, Melbourne Brain Centre, University of Melbourne, Parkville, VIC, Australia;; Department of Neurology, Pavol Jozef Šafárik University in Košice, University Hospital L. Pasteur, Košice, Slovak Republic;; Clinical Operations Global, Boehringer Ingelheim Pharma GmbH & Co. K.G., Biberach, Germany;; Biostatistics and Data Sciences, Boehringer Ingelheim Pharma GmbH & Co. K.G., Ingelheim, Germany;; Department of Neurology, Royal Adelaide Hospital, Adelaide, South Australia, Australia;; Division of Neurology, Stroke Center, University Hospital Basel, Switzerland;; Neurology Service, Hospital de Clínicas de Porto Alegre, Brazil;; Cardiology Medicine, Boehringer Ingelheim International GmbH, Germany;; Department of Neurology, Department of Surgery and Perioperative Care, Seton Dell Medical School Stroke Institute, Austin, TX;; Center of Neurology and Neurosurgery, Goethe University Frankfurt, Frankfurt am Main, Germany;; Department of Neurology with Focus on Neurovascular Diseases and Neurooncology, University of Tübingen, and Hertie Institute for Clinical Brain Research, Germany;; Department of Neurology, Cerebrovaskulární ambulance s.r.o., Brno, Czech Republic;; Department of Neurology, Morsani College of Medicine, University of South Florida, Tampa;; Comprehensive Stroke Center, Department of Neurology, Palacky University, Olomouc, Czech Republic;; Department of Neurology, Evangelisches Klinikum Bethel, Bielefeld, Germany.

**Keywords:** anticoagulants, atrial fibrillation, cardiovascular disease, risk factors, secondary prevention

## Abstract

**Background and Purpose—:**

The RE-SPECT ESUS trial (Randomized, Double-Blind, Evaluation in Secondary Stroke Prevention Comparing the Efficacy and Safety of the Oral Thrombin Inhibitor Dabigatran Etexilate Versus Acetylsalicylic Acid in Patients With Embolic Stroke of Undetermined Source) tested the hypothesis that dabigatran would be superior to aspirin for the prevention of recurrent stroke in patients with embolic stroke of undetermined source. This exploratory subgroup analysis investigates the impact of age, renal function (both predefined), and dabigatran dose (post hoc) on the rates of recurrent stroke and major bleeding.

**Methods—:**

RE-SPECT ESUS was a multicenter, randomized, double-blind trial of dabigatran 150 or 110 mg (for patients aged ≥75 years and/or with creatinine clearance 30 to <50 mL/minute) twice daily compared with aspirin 100 mg once daily. The primary outcome was recurrent stroke.

**Results—:**

The trial, which enrolled 5390 patients from December 2014 to January 2018, did not demonstrate superiority of dabigatran versus aspirin for prevention of recurrent stroke in patients with embolic stroke of undetermined source. However, among the population qualifying for the lower dabigatran dose, the rate of recurrent stroke was reduced with dabigatran versus aspirin (7.4% versus 13.0%; hazard ratio, 0.57 [95% CI, 0.39–0.82]; interaction *P*=0.01). This was driven mainly by the subgroup aged ≥75 years (7.8% versus 12.4%; hazard ratio, 0.63 [95% CI, 0.43–0.94]; interaction *P*=0.10). Stroke rates tended to be lower with dabigatran versus aspirin with declining renal function. Risks for major bleeding were similar between treatments, irrespective of renal function, but with a trend for lower bleeding rates with dabigatran versus aspirin in older patients.

**Conclusions—:**

In subgroup analyses of RE-SPECT ESUS, dabigatran reduced the rate of recurrent stroke compared with aspirin in patients qualifying for the lower dose of dabigatran. These results are hypothesis-generating. Aspirin remains the standard antithrombotic treatment for patients with embolic stroke of undetermined source.

**Registration—:**

URL: https://www.clinicaltrials.gov; Unique identifier: NCT02239120.

Ischemic strokes, according to the TOAST classification (Trial of ORG 10172 in Acute Stroke Treatment), are caused by large-artery extracranial or intracranial atherosclerosis, a cardiac source of embolism, small-artery occlusion, or other less common etiologies.^1^ Twenty to 30% of ischemic strokes are categorized as cryptogenic,^2,3^ which includes patients with an incomplete diagnostic evaluation. The concept of embolic stroke of undetermined source (ESUS) was therefore developed based on the exclusion of lacunar strokes on brain imaging and on the exclusion of known sources for embolism after a required series of diagnostic tests.^4,5^

We conducted the RE-SPECT ESUS study (Randomized, Double-Blind, Evaluation in Secondary Stroke Prevention Comparing the Efficacy and Safety of the Oral Thrombin Inhibitor Dabigatran Etexilate Versus Acetylsalicylic Acid in Patients With Embolic Stroke of Undetermined Source; NCT02239120).^6,7^ Our assumption was that oral anticoagulation with dabigatran would prevent the recurrence of stroke more effectively than aspirin.

The trial did not demonstrate superiority of dabigatran versus aspirin for prevention of recurrent stroke in patients with ESUS. There was no statistically significant difference for major bleeding.^7^ RE-SPECT ESUS was the first Phase III trial in which the dabigatran dose was adapted to the assumed risk of bleeding and pharmacokinetic exposure to dabigatran. Patients aged ≥75 years and/or with creatinine clearance (CrCl) 30 to <50 mL/minute were assigned to the 110 mg twice-daily dose. For stroke prevention in atrial fibrillation (AF), the European label for dabigatran recommends lowering the dose in patients aged ≥80 years and suggests that dose reduction may be considered based on an individual assessment of thromboembolic and bleeding risk in patients aged 75 to 80 years, those with reduced renal function (CrCl 30 to <50 mL/minute), or other patients with an increased bleeding risk.^8^

In RE-SPECT ESUS, we assumed there would be no increase in bleeding risk with dabigatran relative to aspirin when administering the lower dabigatran dose to patients who were elderly and/or renally impaired compared with the standard dose in patients who were younger and/or had normal renal function. In this secondary analysis, we therefore examined the impact of age, renal function, and dabigatran dose on the rates of recurrent stroke and major bleeding.

## Methods

### Data Availability

Qualified scientific and medical researchers may request (via https://vivli.org/) access to de-identified participant study data with documentation describing the structure and content of the datasets. Details are in the Data Supplement.

### Study Design

RE-SPECT ESUS was an international, double-blind, randomized trial,^6,7^ approved by the ethics committees at all participating sites. All patients provided written informed consent.

### Patients

Patients were aged ≥60 years and diagnosed with an ESUS within the previous 3 months, or within the previous 6 months if they had at least 1 additional vascular risk factor, or were aged 18 to 59 years with an ESUS within the previous 3 months and at least 1 additional vascular risk factor. Diagnosis criteria were described previously.^4,7,9^ CrCl <30 mL/minute was an exclusion criterion.

### Treatment

Patients were randomly assigned to dabigatran (150 mg twice daily, or 110 mg twice daily in patients aged ≥75 years or with CrCl 30 to <50 mL/ minute) and aspirin placebo, or aspirin 100 mg once daily and dabigatran placebo (150 or 110 mg twice daily; Figure I in the Data Supplement).

### Outcomes

The primary efficacy outcome was recurrent stroke assessed in a time-to-event analysis. The primary safety outcome was first major bleeding event according to International Society on Thrombosis and Haemostasis criteria.^10^ An independent adjudication committee, blinded to treatment assignment, reviewed and classified the events.

### Statistical Analysis

We summarized clinical characteristics of patients for the baseline subgroups of dabigatran dose, age (<75 or ≥75 years), and CrCl (≥50 or 30 to <50 mL/minute). Age and CrCl were predefined subgroup variables, which we analyzed according to categories relevant to doing decisions. We also analyzed dabigatran dose subgroups, comparing patients assigned to 110 and 150 mg with the matching aspirin patients (those meeting the age and CrCl criteria for dabigatran 110 and 150 mg, respectively).

All analyses followed an intention-to-treat approach. We used Cox proportional hazard models adjusting for stroke or transient ischemic attack before the index stroke (yes/no) to analyze outcomes. For the age analysis, we included renal impairment, and for the renal function analysis, we included the additional variable of age (with categories as above). We provided exploratory *P* values from treatment-by-subgroup tests for interaction. We also analyzed age and renal function as continuous variables for treatment-by-subgroup interaction using analog Cox regression models as described above.

To analyze predictors of major bleeding, we evaluated factors potentially associated with bleeding risk in patients diagnosed with ESUS in the combined treatment groups using descriptive statistics and Cox proportional hazards regression models. We used a univariable Cox regression analysis to identify potential predictors of major bleeding, based on a nominal *P* value <0.05. We built a multivariable Cox regression model based on the set of all potential predictors using backward selection (criteria: *P*<0.1).

## Results

**Figure 1.**
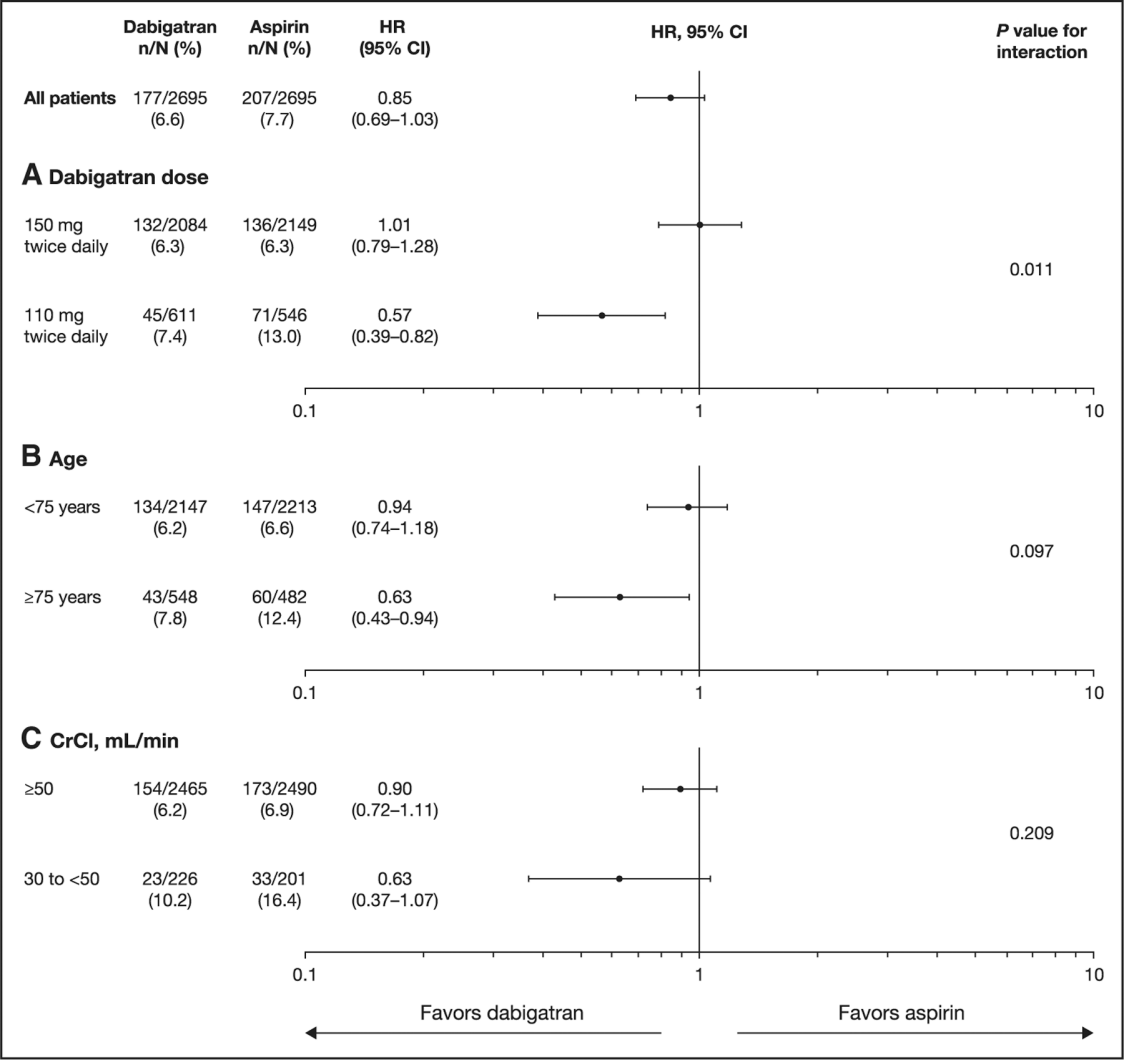
Recurrent stroke with dabigatran vs aspirin by subgroups. Shown by (**A**) dose assignment, (**B**) age, and (**C**) renal function subgroups. Creatinine clearance (CrCl) was estimated by Cockcroft-Gault equation. Patients aged <75 y and with CrCl ≥50 mL/min were randomly assigned to dabigatran 150 mg twice daily plus aspirin placebo or aspirin plus matching dabigatran placebo. Patients aged ≥75 y and/or with CrCl 30 to <50 mL/min were randomly assigned to dabigatran 110 mg twice daily plus aspirin placebo or aspirin plus matching dabigatran placebo.

**Figure 2.**
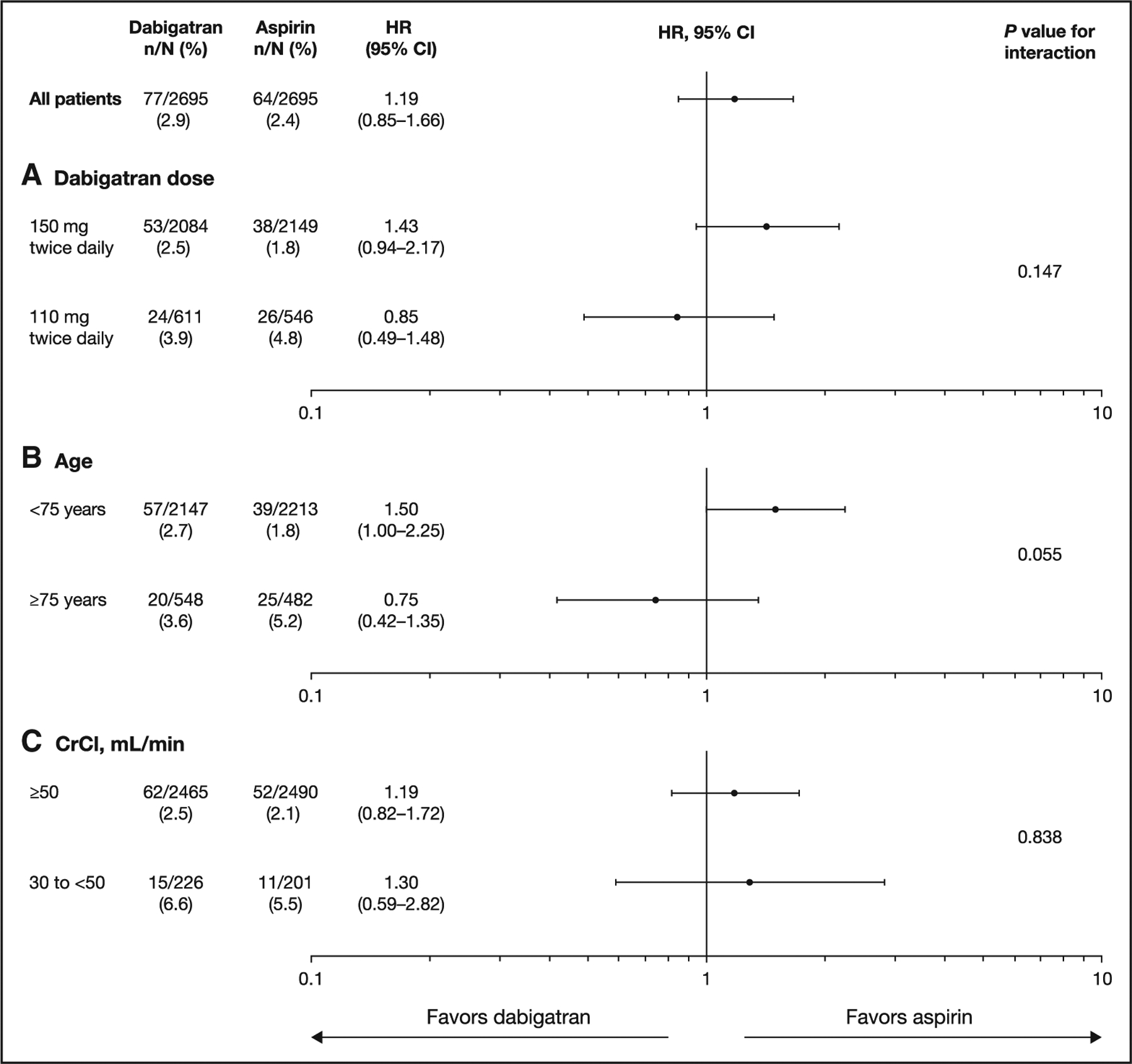
Major bleeding events with dabigatran vs aspirin by subgroups. Shown by (**A**) dose assignment, (**B**) age, and (**C**) renal function subgroups. Creatinine clearance (CrCl) was estimated by Cockcroft-Gault equation. Dabigatran dosing, based on age and CrCl, is described in Figure 1. HR indicates hazard ratio.

Overall, 5390 patients were randomized from December 10, 2014 to January 12, 2018. Median follow-up was 19 months. There were no statistically significant differences between the dabigatran and aspirin groups for the time to first recurrent stroke or for time to first major bleeding in the total study population (Figures 1 and 2).^7^

Baseline demographics for the subgroups according to treatment and dose assignment are shown in the Table, according to age in Table I in the Data Supplement, and CrCl in Table II in the Data Supplement.

Of those patients assigned to dabigatran 110 mg (n=611), 88.2% were aged ≥75 years, 34.9% had CrCl 30 to <50 mL/ minute, and 24.7% were aged ≥75 years with CrCl 30 to <50 mL/minute. The percentage of patients assigned to dabigatran 110 mg only because of older age (ie, age ≥75 years with CrCl ≥50 mL/minute) was 63.5%, and the percentage only because of reduced renal function (ie, CrCl <50 mL/minute with age <75 years) was 10.1%. Patients assigned to dabigatran 110 or 150 mg (n=2084) doses, respectively, had mean ages of 77.9 and 60.6 years, and 49.8% and 33.4% were female (Table). In both treatment groups, patients with CrCl 30 to <50 mL/minute were older than those with CrCl ≥50 mL/ minute (76.7–76.9 versus 62.8–63.4 years), more often female (50.2%–54.0% versus 35.4%–35.6%), and had lower mean body mass index (23.7–23.8 versus 27.5–27.6; Table II in the Data Supplement).

The effects of age, renal function, or dose on the primary outcome of recurrent stroke in patients overall were investigated firstly in a pooled analysis of the dabigatran and aspirin treatment groups together. Overall, recurrent stroke occurred in: 10.0% (6.1%/y) of 1030 patients versus 6.4% (4.1%/y) of 4360 patients aged ≥75 versus <75 years (hazard ratio [HR], 1.26 [95% CI, 0.98–1.62]); 13.1% (8.5%/y) of 427 patients with CrCl 30 to <50 mL/minute versus 6.6% (4.1%/y) of 4955 patients with CrCl ≥50 mL/minute (HR, 1.73 [95% CI, 1.26–2.36]); and 10.0% (6.1%/y) of 1157 patients eligible for dabigatran 110 mg twice daily versus 6.3% (4.0%/y) of 4233 patients eligible for 150 mg twice daily (regardless of actual treatment; HR, 1.52 [95% CI, 1.22–1.89]).

When assessing the treatment effect of dabigatran versus aspirin in the subgroup strata, we observed lower rates of recurrent stroke in patients assigned to dabigatran 110 mg twice daily versus the corresponding aspirin group (patients aged ≥75 years or with CrCl 30 to <50 mL/minute; HR, 0.57 [95% CI, 0.39–0.82]) and similar rates in the patients assigned to dabigatran 150 mg twice daily versus their corresponding aspirin group (HR, 1.01 [95% CI, 0.79–1.28]). In an exploratory analysis based on a nominal alpha level of 5%, these HRs are statistically significantly different between the population qualifying for the lower dose of dabigatran and the population qualifying for the standard dose; interaction *P*=0.011 (Figure 1A). In the subgroup aged ≥75 years, the HR (95% CI) was 0.63 (0.43–0.94) for recurrent stroke in dabigatran- versus aspirin-treated patients, and in those <75 years, it was 0.94 (0.74–1.18); interaction *P*=0.097 (Figure 1B). For patients with CrCl 30 to <50 mL/minute, the HR was 0.63 (0.37–1.07), and for CrCl ≥50, it was 0.90 (0.72–1.11); interaction *P=*0.209 (Figure 1C).

**Figure 3.**
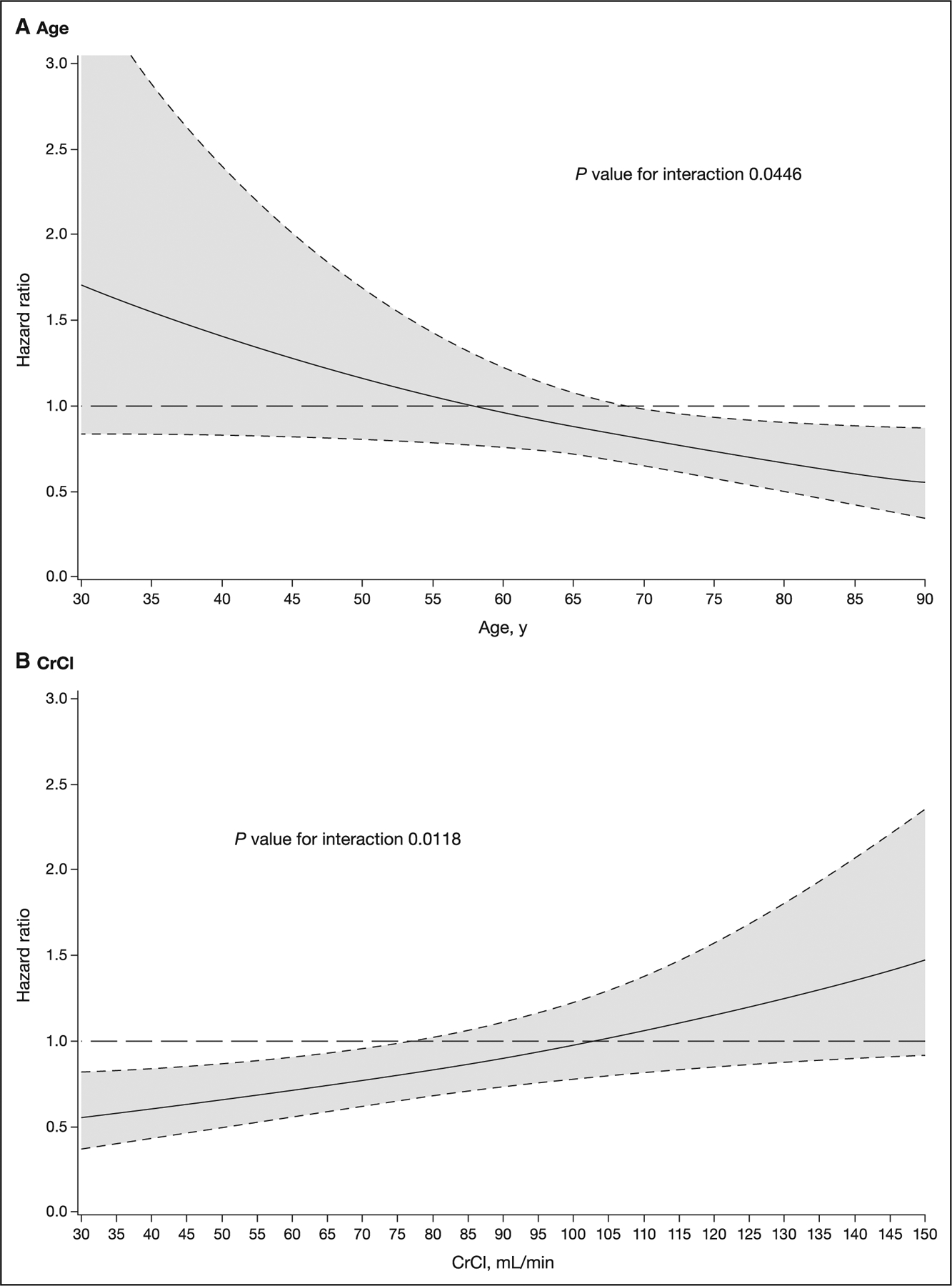
Hazard ratios of recurrent stroke: dabigatran vs aspirin. Shown by (**A**) age and (**B**) estimated creatinine clearance (CrCl). Cox regression models included factors treatment, prior stroke/transient ischemic attack, as well as (**A**) CrCl (≥50 or 30 to <50 mL/min), age, and interaction between age and treatment, or (**B**) age (<75 vs ≥75 y), CrCl, and interaction between CrCl and treatment. Patients with missing data for CrCl are excluded from the analysis model.

Based on the observed trend, we also evaluated age and renal function as continuous variables: rates of stroke were reduced for dabigatran versus aspirin in older patients, and this effect diminished and was even partly reversed for the younger patients (*P* for interaction of treatment with age=0.045; Figure 3A). Those with lower baseline CrCl also had larger treatment benefits with dabigatran versus aspirin than those with higher CrCl who had larger treatment benefit with aspirin (*P* for interaction of treatment with renal function=0.012; Figure 3B). The upper limit of the 95% CI crossed the HR of 1.0 at ≈70 years and at CrCl of ≈75 mL/minute.

Major bleeding in the pooled dabigatran and aspirin treatment groups occurred in: 4.4% (2.6%/y) of 1030 patients aged ≥75 versus 2.2% (1.3%/y) of 4360 patients aged <75 years (HR, 1.48 [95% CI, 0.99–2.20]); 6.1% (3.7%/y) of 427 patients with CrCl 30 to <50 mL/minute versus 2.3% (1.4%/y) of 4955 patients with CrCl ≥50 mL/minute (HR, 2.18 [95% CI, 1.35–3.51]); and 4.3% (2.6%/y) of 1157 patients eligible for dabigatran 110 mg twice daily versus 2.1% (1.3%/y) of 4233 patients eligible for dabigatran 150 mg twice daily (regardless of actual treatment; HR, 1.93 [95% CI, 1.37–2.73]). We observed 25 patients with gastrointestinal major bleeds receiving dabigatran and 17 receiving aspirin.

The treatment effect of dabigatran versus aspirin on major bleeding events was generally consistent with the overall results across the dabigatran dose assignment and renal function subgroups, with nonsignificant interaction *P* values at the 5% level (Figure 2A and 2C). The *P* value for interaction when assessing CrCl as a continuous variable was 0.067. There were trends favoring dabigatran for reduction of major bleeding in the older age group and favoring aspirin in the younger group (interaction *P*=0.055 when assessing age as a categorical variable [Figure 2B] and 0.020 as a continuous variable).

Table III in the Data Supplement shows the patient characteristics that were considered as potential predictors of major bleeding. These are shown for the combined treatment groups (overall study population) according to the occurrence of adjudicated major bleeding and subsequently according to treatment and the occurrence of adjudicated major bleeding. In the combined treatment groups, patients experiencing a major bleed differed from those without major bleeds in terms of age (mean age 69.1 versus 64.1 years; *P*<0.0001) and renal function (median CrCl 69.0 versus 83.0 mL/minute; *P*<0.0001), and by geographic region.

In the univariable Cox regression analysis, based on a nominal *P* value of 0.05, we identified age (HR for 10-year increase, 1.51 [95% CI, 1.28–1.79]; *P*<0.0001) and renal impairment (HR for 10 units of CrCl [mL/minute] increase=0.86 [95% CI, 0.80–0.91]; *P*<0.0001) as predictors of major bleeding, regardless of treatment. Asian region was also an important predictor. Table IV in the Data Supplement shows the full list of variables.

The multivariable Cox regression model derived from backward selection confirmed that age was an important predictor for major bleeding. For every 10-year increase in age, the HR for a major bleed was 1.56 ([95% CI, 1.31–1.84]; *P*<0.0001). Other predictors were region (Asia versus elsewhere: HR, 1.76 [95% CI, 1.24–2.51]; *P*=0.0016) and smoking (current or ex-smoker versus never smoked: HR, 1.33 [95% CI, 0.95–1.87]; *P*=0.0990). Univariable analysis did not reveal this association between smoking status and occurrence of major bleeding, and this result might be an effect of confounding.

## Discussion and Summary

In RE-SPECT ESUS, dabigatran did not significantly reduce recurrent stroke versus aspirin in ESUS patients and did not show a significant difference in major bleeding.^7^ In an exploratory analysis of patients qualifying for a lower dose of dabigatran (110 mg twice daily), who were those aged ≥75 years and/or with reduced renal function, we observed a lower risk of recurrent stroke for those randomized to dabigatran versus aspirin, with a statistically significantly different effect at a nominal alpha level of 5% than in those qualifying for the full dose.

Our original assumption was that patients with ESUS would benefit from anticoagulation over antiplatelet therapy, in part because some might have or develop asymptomatic AF. One of the most important risk factors for AF is age,^11^ and after ESUS, AF is detected by implantable loop recorders substantially more frequently in elderly patients than in younger patients.^12^ Furthermore, in RE-SPECT ESUS, age was shown to be one of the most important predictors for the occurrence of AF during the trial. Patients who developed AF had a higher incidence of recurrent stroke than those who did not develop AF.^13^ This is consistent with our observation of a treatment effect of dabigatran over aspirin in the subgroup of elderly patients as well as in all patients eligible for the lower dose of dabigatran. These patients may be at higher risk of having or developing asymptomatic AF over time, thus leading to a greater effect of the anticoagulant versus aspirin in preventing recurrent stroke. The RE-SPECT ESUS protocol required a dose reduction of dabigatran in patients aged ≥75 years. This group of patients drives the observed effect of the lower dose of dabigatran. To gain more insight into the relationship between age and efficacy of dabigatran, we analyzed age as a continuous variable. Considering the upper 95% CI of the HR (Figure 3A), we observed a reduced risk of recurrent stroke with dabigatran versus aspirin for patients aged >70 years.

Among patients with CrCl 30 to <50 mL/minute, there was a trend for an interaction of a greater treatment effect with the lower dose of dabigatran over aspirin for preventing recurrent stroke. A smaller proportion of patients received the 110 mg twice-daily dose of dabigatran only because their CrCl was <50 mL/minute than only because their age was ≥75 years. Using CrCl as a continuous variable, the upper 95% CI of the HR crossed 1.0 at levels of CrCl <75 mL/minute.

The NAVIGATE ESUS trial (New Approach Rivaroxaban Inhibition of Factor Xa in a Global Trial Versus ASA to Prevent Embolism in Embolic Stroke of Undetermined Source) used a fixed dose of rivaroxaban but did not show a trend for better efficacy of rivaroxaban versus aspirin in elderly or renally impaired patients.^14^ This might be because the observation time was shorter (median 11 months compared with 19 months in RE-SPECT ESUS) because of early termination of the trial for futility, or might be related to the dose of rivaroxaban (15 mg once daily, rather than 20 mg once daily recommended for stroke prevention in patients with AF). Nevertheless, rates of major bleeding were significantly higher with rivaroxaban than with aspirin.

In the RE-LY trial, in which patients were randomly assigned to receive fixed dabigatran doses, increasing age was an important predictor of major bleeding with dabigatran versus warfarin.^15,16^ The dabigatran labels, except in the United States, recommend a lower dose for patients aged ≥80 years, and state a reduced dose should be considered in patients aged 75 to 80 years.^8^ Our assumption that bleeding risk would not be increased with dabigatran versus aspirin in elderly patients, when the dose was reduced in this group, compared with that in younger patients receiving the standard dose, was supported in RE-SPECT ESUS. As in the overall trial population, patients aged ≥75 years and/or with CrCl 30 to <50 mL/minute had similar rates of major bleeding with dabigatran and aspirin. There was a trend toward less bleeding with dabigatran versus aspirin in patients aged ≥75 years. However, independent of treatment, age and renal function were significant predictors for major bleeding in a univariable analysis of predictors. Age remained a significant predictor in the multivariate analysis. Although the annualized rate of major bleeding with aspirin in elderly patients was higher than reported in some studies, and higher than found in the overall trial, it was consistent with previous reports of an increased risk of major bleeding with aspirin in older patients.^17,18^

Of note, the analyses presented are based on an overall clinically neutral trial, which failed to show superiority for its primary end point. Two of the subgroup analyses (age and renal function) were prespecified, whereas analysis by dabigatran dose was post hoc. The power to detect a treatment effect is limited, as the study was designed to test the total effect across subgroups, and false-negative and false-positive effects could arise due to testing of multiple subgroups. The subgroup analyses presented are therefore hypothesis-generating and should not result in modified treatment recommendations for patients with ESUS.

## Conclusions

At present, with 2 studies (RE-SPECT ESUS and NAVIGATE ESUS) that failed to show superior efficacy of a nonvitamin K antagonist oral anticoagulant compared with aspirin in patients with ESUS, the standard of care for secondary stroke prevention remains aspirin.^7,14^ Our findings suggest considering a more intense search for cardioembolism or asymptomatic AF in ESUS patients, and the elderly in particular,^19^ and then starting anticoagulation upon diagnosis. Also, recruiting patients with a higher probability of developing AF, identified by echocardiographic parameters or biomarkers (as in the ARCADIA (Atrial Cardiopathy and Antithrombotic Drugs in Prevention After Cryptogenic Stroke) and ATTICUS (Apixaban for Treatment of Embolic Stroke of Undetermined Source) trials, which compare apixaban with aspirin in selected ESUS patients),^19,20^ might be an option to more successfully explore superiority for recurrent stroke prevention of a nonvitamin K antagonist oral anticoagulant over aspirin in patients with ESUS.

## Supplementary Material

RE SPECT ESUS Suppl

## Figures and Tables

**Table.** Baseline Characteristics of Patients According to Treatment and Dose Assignment

	Dabigatran 1150 mg Twice Daily*	Dabigatran 110 mg Twice Daily†	Aspirin (Dabigatran 150 mg Twice Daily Comparator Group)*	Aspirin (Dabigatran 110 mg Twice Daily Comparator Group)†
Patients, n (%)	2084 (100)	611 (100)	2149 (100)	546 (100)
Age, y; mean (SD)	60.6 (9.7)	77.9 (5.3)	60.4 (9.7)	77.8 (5.1)
Female, n (%)	697 (33.4)	304 (49.8)	745 (34.7)	241 (44.1)
Region, n (%)				
Non-Asia	1614 (77.4)	465 (76.1)	1711 (79.6)	402 (73.6)
Asia	470 (22.6)	146 (23.9)	438 (20.4)	144 (26.4)
Body mass index, kg/m^2^; mean (SD)	27.6 (5.2)	25.9 (4.2)	27.7 (5.1)	25.8 (4.2)
Renal function (CrCl), mL/min; median (IQR)	89.0 (73.0–111.0)	56.0 (46.0–67.0)	91.0 (75.0–113.0)	56.0 (46.7–68.3)
Diabetes mellitus, n (%)	471 (22.6)	114 (18.7)	516 (24.0)	123 (22.5)
Prior stroke/transient ischemic attack, n (%)	340 (16.3)	135 (22.1)	380 (17.7)	120 (22.0)
Hypertension, n (%)	1509 (72.4)	487 (79.7)	1551 (72.2)	434 (79.5)
NIHSS score, median (IQR)	1.0 (0.0–2.0)	1.0 (0.0–2.0)	1.0 (0.0–2.0)	1.0 (0.0–2.0)
Prior MBE or predisposition to bleeding, n (%)	8 (0.4)	1 (0.2)	6 (0.3)	4 (0.7)
Smoking, n (%)				
Never smoked	885 (42.5)	384 (62.8)	909 (42.3)	302 (55.3)
Current or ex-smoker	1198 (57.5)	227 (37.2)	1240 (57.7)	244 (44.7)
Proton pump inhibitor at baseline, n (%)	619 (29.7)	257 (42.1)	607 (28.2)	220 (40.3)
NSAID/cyclooxygenase-II inhibitor at baseline, n (%)	98 (4.7)	39 (6.4)	98 (4.6)	38 (7.0)
Antiplatelets at baseline, n (%)	584 (28.0)	168 (27.5)	641 (29.8)	170 (31.1)
Optional use of aspirin for CAD (assessed at baseline), n (%)‡	133 (6.4)	36 (5.9)	106 (4.9)	32 (5.9)
Time from index stroke to randomization, d				
<8	63 (3.0)	32 (5.2)	78 (3.6)	19 (3.5)
8–30	670 (32.1)	222 (36.3)	732 (34.1)	205 (37.5)
31–90	996 (47.8)	223 (36.5)	994 (46.3)	206 (37.7)
≥91	355 (17.0)	134 (21.9)	344 (16.0)	116 (21.2)

Body mass index, renal function, NIHSS score, smoking, and time from index stroke to randomization were missing in 41, 5, 11, 1, and 1 patients, respectively. CrCl was estimated by Cockcroft-Gault equation. CAD indicates coronary artery disease; CrCl, creatinine clearance; IQR, interquartile range; MBE, major bleeding event; NIHSS, National Institutes of Health Stroke Scale; and NSAID, nonsteroidal anti-inflammatory drug.

* Patients aged <75 y and with CrCl ≥50 mL/min were randomly assigned to dabigatran 150 mg twice daily plus aspirin placebo or aspirin plus matching dabigatran placebo.

† Patients aged ≥75 y and/or with CrCl 30 to <50 mL/min were randomly assigned to dabigatran 110 mg twice daily plus aspirin placebo or aspirin plus matching dabigatran placebo.

‡ Patients with CAD could receive optional add-on aspirin (if randomized to dabigatran) or placebo (if randomized to aspirin).
