# Supplementary material for: Antithrombotic Treatment of Embolic Stroke of Undetermined Source RE-SPECT ESUS Elderly and Renally Impaired Subgroups
Source: Stroke. Author manuscript; Available in PMC 2020 Jul 24. (PMC7379165; doi:10.1161/STROKEAHA.119.028643)
Supplement: RE SPECT ESUS Suppl [file NIHMS1608671-supplement-RE_SPECT_ESUS_Suppl.pdf]

SUPPLEMENTAL MATERIAL

RE-SPECT ESUS: Subgroup Analyses

Antithrombotic Treatment of Embolic Stroke of Undetermined Source: RE-SPECT ESUS Elderly and Renally Impaired Subgroups

Hans-Christoph Diener, MD; Ralph L. Sacco, MD; J. Donald Easton, MD; Christopher B. Granger, MD; Michal Bar, PhD; Richard A. Bernstein, MD; Michael Brainin, MD; Martina Brueckmann, MD; Lisa Cronin, MD; Geoffrey Donnan, MD; Zuzana Gdovinová, MD; Claudia Grauer, PhD; Eva Kleine, MSc; Timothy J. Kleinig, PhD; Philippe Lyrer, MD; Sheila Martins, PhD; Juliane Meyerhoff, PhD; Truman Milling, MD; Waltraud Pfeilschifter, MD; Sven Poli, MD; Michal Reif, MD; David Z. Rose, MD; Daniel Šaňák, MD; Wolf-Rüdiger Schäbitz, MD

From the Faculty of Medicine, Institute for Medical Informatics, Biometry and Epidemiology, University Duisburg-Essen, Essen, Germany (H-C.D.); Clinical and Translational Science, Miller School of Medicine, University of Miami, Miami, Florida (R.L.S.); Department of Neurology, University of California, San Francisco, California (J.D.E.); Duke Clinical Research Institute, Duke University Medical Center, Durham, North Carolina (C.B.G.); Department of Neurology, University Hospital Ostrava, Ostrava-Poruba-Poruba, Czech Republic (M.Bar); Department of Neurology, Northwestern University, Chicago, Illinois (R.A.B.); Department of Neurosciences and Preventive Medicine, Danube University Krems, Krems an der Donau, Austria (M.Brainin); Metabolism Medicine, Boehringer Ingelheim International GmbH, Ingelheim, Germany (M.Brueckmann.); Faculty of Medicine Mannheim of the University of Heidelberg, Mannheim, Germany (M.Brueckmann.); Cardiometabolic Medicine, Boehringer Ingelheim Ltd, Burlington, Ontario, Canada (L.C.); Department of Neurology, Melbourne Brain Centre, University of Melbourne, Parkville, Victoria, Australia (G.D.); Department of Neurology, Pavol Jozef Šafárik University in Košice, University Hospital L. Pasteur, Košice, Slovak Republic (Z.G.); Clinical Operations Global, Boehringer Ingelheim Pharma GmbH & Co. K.G., Biberach, Germany (C.G.); Biostatistics and Data Sciences, Boehringer Ingelheim Pharma GmbH & Co. K.G., Ingelheim, Germany (E.K.); Department of Neurology, Royal Adelaide Hospital, Adelaide, South Australia, Australia (T.J.K.); Division of Neurology, Stroke Center, University Hospital Basel, Basel, Switzerland (P.L.); Neurology Service, Hospital de Clínicas de Porto Alegre, Porto Alegre, Brazil (S.M.); Cardiology Medicine, Boehringer Ingelheim International GmbH, Ingelheim, Germany (J.M.); Department of Neurology, Department of Surgery and Perioperative Care, Seton Dell Medical School Stroke Institute, Austin, Texas (T.M.); Center of Neurology and Neurosurgery, Goethe University Frankfurt, Frankfurt am Main, Germany (W.P.); Department of Neurology with Focus on Neurovascular Diseases and Neurooncology, University of Tübingen, and Hertie Institute for Clinical Brain Research, Tübingen, Germany (S.P.); Department of Neurology, Cerebrovaskulární ambulance s.r.o., Brno, Czech Republic (M.R.); Department of Neurology, Morsani College of Medicine, University of South Florida, Tampa, Florida (D.Z.R.); Comprehensive Stroke Center,

Department of Neurology, Palacky University, Olomouc, Czech Republic (D.S.); Department of Neurology, Evangelisches Klinikum Bethel, Bielefeld, Germany (W-R.S.).

Correspondence to:
Prof. Hans-Christoph Diener, Faculty of Medicine, Institute for Medical Informatics, Biometry and Epidemiology, University Duisburg-Essen, Hufelandstrasse 55, 45147, Essen, Germany
Tel: +49 201 723 6540
h.diener@uni-essen.de, hans.diener@uk-essen.de

SUPPLEMENTAL FIGURES

Supplemental Figure I. Trial design.

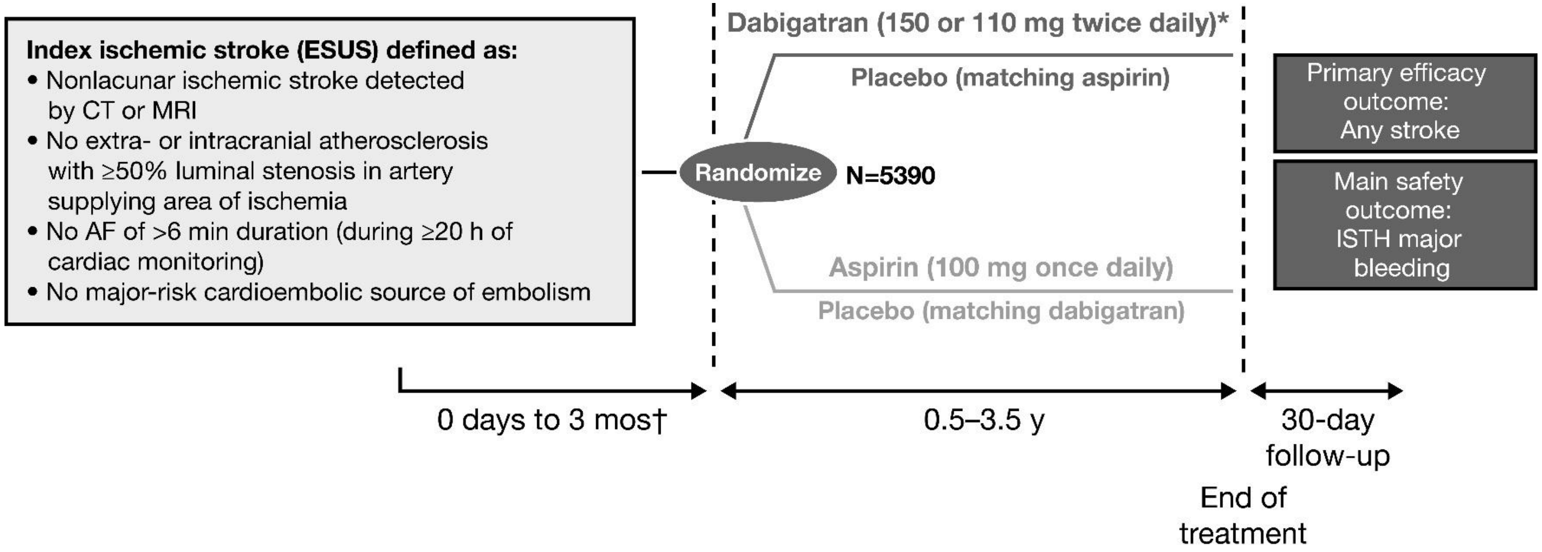

*Dabigatran 110 mg twice daily for patients aged ≥75 years and/or with creatinine clearance 30 to <50 mL/min.
†0 days to 6 months in patients aged ≥60 years with additional risk factors.

- If patients who initially received dabigatran 150 mg twice daily experienced a deterioration in creatinine clearance to 30 to <50 mL/min, a gastrointestinal bleed, or turned 75 years old during the trial, their dose was reduced to 110 mg twice daily.
- Patients with coronary artery disease could receive aspirin if they were assigned to the dabigatran group, or placebo if they were assigned to the aspirin group, supplied in a blinded manner.
- Treatment was planned for 6 months (minimum) to 3.5 years (maximum).

AF indicates atrial fibrillation; CT, computed tomography; ESUS, embolic stroke of undetermined source; ISTH, International Society on Thrombosis and Haemostasis; mos, months; MRI, magnetic resonance imaging.

SUPPLEMENTAL TABLES

Supplemental Table I. Baseline Characteristics of Patients According to Age Subgroups

	**Dabigatran by Age**		**Aspirin by Age**	
	**<75 y***	**≥75 y†**	**<75 y***	**≥75 y†**
Patients, n (%)	2147 (100)	548 (100)	2213 (100)	482 (100)
Age, mean (SD), y	60.8 (9.6)	79.2 (3.6)	60.6 (9.7)	79.0 (3.3)
Female, n (%)	734 (34.2)	267 (48.7)	765 (34.6)	221 (45.9)
Region, n (%)				
Non-Asia	1647 (76.7)	432 (78.8)	1738 (78.5)	375 (77.8)
Asia	500 (23.3)	116 (21.2)	475 (21.5)	107 (22.2)
BMI, mean (SD), kg/m^2	27.4 (5.2)	26.2 (4.0)	27.6 (5.1)	26.1 (4.2)
Renal function (CrCl), median (IQR), mL/min	88.3 (71.0–110.0)	58.0 (48.0–68.0)	89.7 (73.0–112.0)	59.0 (48.0–70.0)
CrCl 30 to <50 mL/min, n (%)	75 (3.5)	151 (27.6)	61 (2.8)	140 (29.0)
Diabetes mellitus, n (%)	486 (22.6)	99 (18.1)	537 (24.3)	102 (21.2)
Prior stroke/TIA, n (%)	356 (16.6)	119 (21.7)	396 (17.9)	104 (21.6)
Hypertension, n (%)	1558 (72.6)	438 (79.9)	1603 (72.4)	382 (79.3)
NIHSS score, median (IQR)	1 (0–2)	1 (0–2)	1 (0–2)	1 (0–2)
Prior MBE or predisposition to bleeding, n (%)	8 (0.4)	1 (0.2)	6 (0.3)	4 (0.8)

Smoking, n (%)				
Never smoked	919 (42.8)	350 (63.9)	939 (42.4)	272 (56.4)
Current or ex-smoker	1227 (57.1)	198 (36.1)	1274 (57.6)	210 (43.6)
PPI at baseline, n (%)	648 (30.2)	228 (41.6)	641 (29.0)	186 (38.6)
NSAID/COX-II inhibitor at baseline, n (%)	104 (4.8)	33 (6.0)	103 (4.7)	33 (6.8)
Antiplatelets at baseline, n (%)	602 (28.0)	150 (27.4)	652 (29.5)	159 (33.0)
Optional use of aspirin for CAD (assessed at baseline), n (%)*	136 (6.3)	33 (6.0)	112 (5.1)	26 (5.4)
Dabigatran dose, n (%)				
110 mg twice daily	72 (3.4)	539 (98.4)	69 (3.1)	477 (99.0)
150 mg twice daily	2075 (96.6)	9 (1.6)†	2144 (96.9)	5 (1.0)†
Time from index stroke to randomization, days, n (%)				
<8	64 (3.0)	31 (5.7)	79 (3.6)	18 (3.7)
8−30	691 (32.2)	201 (36.7)	753 (34.0)	184 (38.2)
31−90	1021 (47.6)	198 (36.1)	1020 (46.1)	180 (37.3)
≥91	371 (17.3)	118 (21.5)	360 (16.3)	100 (20.7)

BMI indicates body mass index; CAD, coronary artery disease; COX, cyclooxygenase; CrCl, creatinine clearance; IQR, interquartile range; MBE, major bleeding event; NIHSS, National Institutes of Health Stroke Scale; NSAID, nonsteroidal anti-inflammatory drug; PPI, proton pump inhibitor; TIA, transient ischemic attack; and SD, standard deviation.

*Patients aged <75 years and with CrCl ≥50 mL/min were randomly assigned to dabigatran 150 mg twice daily plus aspirin placebo or aspirin plus dabigatran 150 mg twice daily placebo.

†Patients aged ≥75 years and/or with CrCl 30 to <50 mL/min were randomly assigned to dabigatran 110 mg twice daily plus aspirin placebo or aspirin plus dabigatran 110 mg twice daily placebo.

‡Patients with CAD could receive optional add-on aspirin (if randomized to dabigatran) or placebo (if randomized to aspirin).

BMI was missing in 41 patients overall; renal function, NIHSS score, smoking, and time from index stroke to randomization were missing in 5, 11, 1, and 1 patients, respectively.

CrCl was estimated by Cockcroft−Gault equation.

Supplementary Table II. Baseline Characteristics of Patients According to Renal Function Subgroups

	Dabigatran by CrCl		Aspirin by CrCl	
	**30 to <50 mL/min**	**≥50 mL/min**	**30 to <50 mL/min**	**≥50 mL/min**
Patients, n (%)	226 (100)	2465 (100)	201 (100)	2490 (100)
Age, mean (SD), y	76.7 (7.4)	63.4 (11.1)	76.9 (7.5)	62.8 (11.0)
Age ≥75 years, n (%)	151 (66.8)	395 (16.0)	140 (69.7)	340 (13.7)
Female, n (%)	122 (54.0)	878 (35.6)	101 (50.2)	882 (35.4)
Region, n (%)				
Non-Asia	146 (64.6)	1929 (78.3)	116 (57.7)	1993 (80.0)
Asia	80 (35.4)	536 (21.7)	85 (42.3)	497 (20.0)
BMI, mean (SD), kg/m^2	23.8 (3.6)	27.5 (5.0)	23.7 (3.5)	27.6 (5.0)
Renal function (CrCl), median (IQR), mL/min	43.0 (38.0–46.0)	85.0 (68.0–106.6)	44.0 (40.0–47.0)	87.0 (70.0–109.0)
Diabetes mellitus, n (%)	43 (19.0)	541 (21.9)	47 (23.4)	591 (23.7)
Prior stroke/TIA, n (%)	56 (24.8)	419 (17.0)	48 (23.9)	451 (18.1)
Hypertension, n (%)	185 (81.9)	1807 (73.3)	161 (80.1)	1820 (73.1)
NIHSS score, median (IQR)	1 (0–2)	1 (0–2)	1 (0–2)	1 (0–2)
Prior MBE or predisposition to bleeding, n (%)	0	9 (0.4)	0	9 (0.4)
Smoking, n (%)				

Never smoked	122 (54.0)	1144 (46.4)	118 (58.7)	1090 (43.8)
Current or ex-smoker	104 (46.0)	1320 (53.5)	83 (41.3)	1400 (56.2)
PPI at baseline, n (%)	93 (41.2)	782 (31.7)	89 (44.3)	737 (29.6)
NSAID/COX-II inhibitor at baseline, n (%)	13 (5.8)	124 (5.0)	16 (8.0)	120 (4.8)
Antiplatelets at baseline, n (%)	62 (27.4)	689 (28.0)	53 (26.4)	757 (30.4)
Optional use of aspirin for CAD (assessed at baseline), n (%)*	17 (7.5)	152 (6.2)	7 (3.5)	130 (5.2)
Dabigatran dose, n (%)				
110 mg twice daily	213 (94.2)	396 (16.1)	195 (97.0)	349 (14.0)
150 mg twice daily	13 (5.8)†	2069 (83.9)	6 (3.0)†	2141 (86.0)
Time from index stroke to randomization, days				
<8	7 (3.1)	88 (3.6)	4 (2.0)	93 (3.7)
8−30	87 (38.5)	802 (32.5)	81 (40.3)	854 (34.3)
31−90	89 (39.4)	1129 (45.8)	74 (36.8)	1124 (45.1)
≥90	43 (19.0)	446 (18.1)	42 (20.9)	418 (16.8)

BMI indicates body mass index; CAD, coronary artery disease; COX, cyclooxygenase; CrCl, creatinine clearance; IQR, interquartile range; MBE, major bleeding event; NIHSS, National Institutes of Health Stroke Scale; NSAID, nonsteroidal anti-inflammatory drug; PPI, proton pump inhibitor; TIA, transient ischemic attack; and SD, standard deviation.

*Patients with CAD could receive optional add-on aspirin (if randomized to dabigatran) or placebo (if randomized to aspirin).

†Thirteen and 6 patients were assigned to different CrCl categories by the interactive voice response system versus the remote data capture system.

Eight patients were excluded from the analysis because information on CrCl was missing or CrCl was <30 mL/min. BMI was missing in 41 patients overall; renal function, NIHSS score, smoking, and time from index stroke to randomization were missing in 5, 11, 1, and 1 patients, respectively.

CrCl was estimated by Cockcroft−Gault equation.

Supplementary Table III. Baseline Characteristics of Patients According to the Occurrence of Adjudicated Major Bleeding (Randomized Set)

	**Overall, n (%)**			**Dabigatran, n (%)**		**Aspirin, n (%)**	
	**No Major Bleed**	**Major Bleed**	***P* Value***	**No Major Bleed**	**Major Bleed**	**No Major Bleed**	**Major Bleed**
Patients, n (%)	5249 (100)	141 (100)		2618 (100)	77 (100)	2631 (100)	64 (100)
Age, mean (SD), y	64.1 (11.4)	69.1 (10.7)	<0.0001†	64.5 (11.4)	67.5 (11.4)	63.7 (11.4)	71.1 (9.6)
Female sex, n (%)	1936 (36.9)	51 (36.2)	0.8625	967 (36.9)	34 (44.2)	969 (36.8)	17 (26.6)
Region, n (%)			0.0026				
Non-Asia	4097 (78.1)	95 (67.4)		2026 (77.4)	53 (68.8)	2071 (78.7)	42 (65.6)
Asia	1152 (21.9)	46 (32.6)		592 (22.6)	24 (31.2)	560 (21.3)	22 (34.4)
BMI, mean (SD), kg/m^2	27.3 (5.0)	26.5 (5.3)	0.1068†	27.2 (5.0)	26.5 (6.2)	27.3 (5.0)	26.6 (3.8)
Renal function (CrCl), median (IQR), mL/min	83.0 (65.0–106.0)	69.0 (53.3–88.4)	<0.0001†	82.0 (64.0–105.0)	71.0 (54.0–91.0)	85.0 (67.0–107.0)	68.0 (52.5–85.0)
Diabetes mellitus, n (%)	1184 (22.6)	40 (28.4)	0.1040	564 (21.5)	21 (27.3)	620 (23.6)	19 (29.7)
Prior stroke/TIA, n (%)	943 (18.0)	32 (22.7)	0.1499	461 (17.6)	14 (18.2)	482 (18.3)	18 (28.1)
Hypertension, n (%)	3878 (73.9)	103 (73.0)	0.8246	1944 (74.3)	52 (67.5)	1934 (73.5)	51 (79.7)
NIHSS score, median (IQR)	1 (0–2)	0 (0–2)	0.3482†	1 (0–2)	1 (0–2)	1 (0–2)	0 (0–2)

Prior MBE or predisposition to bleeding, n (%)	18 (0.3)	1 (0.7)	0.4689	9 (0.3)	0	9 (0.3)	1 (1.6)
Smoking, n (%)			0.4027				
Never smoked	2420 (46.1)	60 (42.6)		1237 (47.2)	32 (41.6)	1183 (45.0)	28 (43.8)
Current or ex-smoker	2828 (53.9)	81 (57.4)		1380 (52.7)	45 (58.4)	1448 (55.0)	36 (56.3)
PPI at baseline, n (%)	1655 (31.5)	48 (34.0)	0.5265	852 (32.5)	24 (31.2)	803 (30.5)	24 (37.5)
NSAID/COX−II inhibitor at baseline, n (%)	262 (5.0)	11 (7.8)	0.1332	134 (5.1)	3 (3.9)	128 (4.9)	8 (12.5)
Antiplatelets at baseline, n (%)	1513 (28.8)	50 (35.5)	0.0866	723 (27.6)	29 (37.7)	790 (30.0)	21 (32.8)
Treatment, n (%)			0.2673				
Aspirin	2631 (50.1)	64 (45.4)		–	–	–	–
Dabigatran	2618 (49.9)	77 (54.6)		–	–	–	–
Dabigatran dose, n (%)							
110 mg twice daily	–	–		587 (22.4)	24 (31.2)	520 (19.8)	26 (40.6)
150 mg twice daily	–	–		2031 (77.6)	53 (68.8)	2111 (80.2)	38 (59.4)

BMI indicates body mass index; COX, cyclooxygenase; CrCl, creatinine clearance; IQR, interquartile range; MBE, major bleeding event; NIHSS, National Institutes of Health Stroke Scale; NSAID, nonsteroidal anti-inflammatory drug; PPI, proton pump inhibitor; TIA, transient ischemic attack; and SD, standard deviation.

*All *P* values from chi-square test (unless shown otherwise) comparing patients with versus without major bleeding in the overall population.
†From t-test comparing patients with versus without major bleeding in the overall population.

BMI was missing in 41 patients overall; renal function, NIHSS score and smoking were missing in 5, 11, and 1 patients, respectively.
CrCl was estimated by Cockcroft−Gault equation.

Supplementary Table IV. Univariable Analysis of Predictors of Major Bleeding

	Univariable Analysis	
	**HR (95% CI)**	***P* value**
Treatment (dabigatran vs aspirin)	1.20 (0.86–1.68)	0.2738
Age, y (10 units increase)	1.51 (1.28–1.79)	<0.0001
Sex (male vs female)	1.04 (0.74–1.47)	0.8183
Region (Asia vs non-Asia)	1.75 (1.23–2.49)	0.0018
BMI, kg/m^2 (5 units increase)	0.86 (0.72–1.02)	0.0906
CrCl, mL/min (10 units increase)	0.86 (0.80–0.91)	<0.0001
Diabetes mellitus (yes vs no)	1.36 (0.94–1.96)	0.1015
Prior stroke/TIA (yes vs no)	1.33 (0.90–1.98)	0.1540
Hypertension (yes vs no)	0.96 (0.66–1.39)	0.8143
NIHSS score (1 unit increase)	0.97 (0.89–1.06)	0.4790
Prior MBE or predisposition to bleeding (yes vs no)	1.92 (0.27–13.70)	0.5166
Smoking (ex/current vs never smoker)	1.15 (0.82–1.61)	0.4069
PPI at baseline (yes vs no)	1.09 (0.77–1.54)	0.6397
NSAID/COX-II inhibitor at baseline (yes vs no)	1.61 (0.87–2.98)	0.1285
Antiplatelets at baseline (yes vs no)	1.31 (0.93–1.85)	0.1226

BMI indicates body mass index; CI, confidence interval; COX, cyclooxygenase; CrCl, creatinine clearance; HR, hazard ratio; MBE, major bleeding event; NIHSS, National Institutes of Health Stroke Scale; NSAID, nonsteroidal anti-inflammatory drug; PPI, proton pump inhibitor; TIA, transient ischemic attack; and SD, standard deviation.

Patients with missing categories or missing values for a continuous variable were not considered for univariate regression analysis in the corresponding variable.

CrCl was estimated by Cockcroft−Gault equation.

Data Sharing

To ensure independent interpretation of clinical study results, Boehringer Ingelheim grants all external authors access to all relevant material, including participant-level clinical study data, and relevant material as needed by them to fulfill their role and obligations as authors under the ICMJE criteria.

Furthermore, clinical study documents (eg, study report, study protocol, statistical analysis plan) and participant clinical study data are available to be shared after publication of the primary manuscript in a peer-reviewed journal and if regulatory activities are complete and other criteria met per the BI Policy on Transparency and Publication of Clinical Study Data: https://trials.boehringer-ingelheim.com/transparency_policy.html

Prior to providing access, documents will be examined, and, if necessary, redacted and the data will be de-identified, to protect the personal data of study participants and personnel, and to respect the boundaries of the informed consent of the study participants.

Clinical Study Reports and Related Clinical Documents can be requested via this link: https://trials.boehringer-ingelheim.com/trial_results/clinical_submission_documents.html

All such requests will be governed by a Document Sharing Agreement.

Bona fide, qualified scientific and medical researchers may request access to de-identified participant study data with corresponding documentation describing the structure and content of the datasets. Upon approval, and governed by a Data Sharing Agreement, data are shared for a period of 1 year, which may be extended upon request.

Researchers should use https://vivli.org/ to request access to study data.
